# Predictors of exclusive breastfeeding and consumption of soft, semi-solid or solid food among infants in Boucle du Mouhoun, Burkina Faso: A cross-sectional survey

**DOI:** 10.1371/journal.pone.0179593

**Published:** 2017-06-22

**Authors:** Jenny A. Cresswell, Rasmané Ganaba, Sophie Sarrassat, Simon Cousens, Henri Somé, Abdoulaye Hama Diallo, Veronique Filippi

**Affiliations:** 1 London School of Hygiene & Tropical Medicine, London, United Kingdom; 2 AFRICSanté, Bobo-Dioulasso, Burkina Faso; 3 Centre MURAZ, Bobo-Dioulasso, Burkina Faso; TNO, NETHERLANDS

## Abstract

**Introduction:**

Exclusive breastfeeding is among the most effective interventions for preventing child mortality. The objectives of this paper are to describe infant feeding knowledge and practices in Boucle du Mouhoun, Burkina Faso; to identify predictors of exclusive breastfeeding among infants <6 months, and consumption of soft, semi-solid or solid food among infants 6–11 months; to describe mothers’ sources of information regarding breastfeeding.

**Methods:**

A cross-sectional survey (n = 2288) of a representative sample of women aged 15–49 years with at least one live birth in past year took place during June and July 2015. Crude and multivariable random-effects logistic regressions were used to identify factors predictive of exclusive breastfeeding and consumption of soft, semi-solid or solid food.

**Results:**

30% of infants <6 months were exclusively breastfed; 67% of infants age 6–11 months consumed soft, semi-solid or solid food the day and night before the interview. 2% of infants age 6–11 months had a minimum acceptable diet. There was strong evidence of a positive association between knowledge and practice of exclusive breastfeeding, nonetheless 60% of mothers who correctly identified that an infant should be exclusively breastfed for 6 months did not breastfeed their infant exclusively. Only 42% of mothers reported receiving advice on breastfeeding from a health worker, despite all mothers having contact with a health worker at least once during pregnancy or postpartum.

**Conclusion:**

Given poor practices and low levels of knowledge, targeted interventions are needed to improve infant nutrition in Boucle du Mouhoun during antenatal, delivery and postnatal care. Most women now deliver in a facility in Burkina Faso; increased attention should be paid to ensuring that existing guidelines relating to support and counselling for infant feeding are adhered to. Factors such as social norms are also important and these should be investigated in more detail using qualitative methods.

## Introduction

Exclusive breastfeeding among infants under 6 months, and continued breastfeeding accompanied by appropriate complementary feeding among infants 6 months or older, have been estimated to be among the most effective interventions for preventing deaths in children under five years globally [1–3]. In addition, breastfeeding has long-term benefits to child health and development such as protection against child infections, increased performance in intelligence tests, and likely reductions in overweight and diabetes [3]. Consequently, the World Health Organization (WHO) recommends that the optimal way of feeding infants is exclusive breastfeeding up until 6 months of age, with continued breastfeeding alongside complementary feeding up to two years or beyond [4].

In Burkina Faso, neonatal and infant mortality rates are high; estimated at 20, 9, and 32 deaths per 1000 live births during the early neonatal, late neonatal and post-neonatal periods respectively [5,6]. Only around one in four infants under 6 months are exclusively breastfed and complementary feeding indicators are poor [7,8]. In West Africa, not breastfeeding exclusively has been shown to be associated with lower levels of education, the infant having contracted diarrhoea in the past two weeks, lack of antenatal care, and delivery assisted by traditional birth assistants [9]. However, a mother’s decision to breastfeed is based on many different factors, and different characteristics and behaviours are associated with breastfeeding behaviours across different settings depending on context [10–13]. Furthermore, indicators of different breastfeeding behaviours (such as exclusive breastfeeding, continued breastfeeding or early initiation of breastfeeding) have been found to be only moderately correlated [3].

Evidence-based interventions are urgently needed to improve infant and young child feeding practices [14]. Promotion of exclusive breastfeeding during six individual home visits by peer counsellors was shown to be successful in more than doubling mothers-reported exclusive breastfeeding rates in a cluster-randomised trial conducted in Banfora, south-west Burkina Faso [15,16]. This intervention was resource-intensive: cost-effectiveness data from Burkina Faso have not been published, however the intervention when implemented in Uganda as part of the same trial found that it was not likely to be cost-effective in averting disability-adjusted life years in that setting [17].

A repeated cross-sectional cluster-randomised trial is currently underway in the Boucle du Mouhoun region of Burkina Faso, evaluating components of the Alive & Thrive program model (http://aliveandthrive.org/), specifically training and supporting healthcare workers to deliver high-quality counselling in facilities, training of community health workers to conduct targeted home visits to improve infant and young child feeding practices, and community mobilisation activities with the aim of shifting social norms towards optimal breastfeeding practices. Descriptive findings from data collected during the baseline survey for this trial are presented in this paper; the trial will conclude with an endline survey in 2017.

The objectives of this paper are to: (1) describe key infant feeding knowledge and practices in Boucle du Mouhoun; (2) identify predictors of exclusive breastfeeding among infants less than 6 months old, and of consuming soft, semi-solid or solid food the day and night before the interview among infants aged 6 to 11 months old; (3) describe where mothers receive information and advice relating to breastfeeding.

## Methods

### Setting

Boucle du Mouhoun is one of the 13 regions of Burkina Faso, located in the north-west of the country. The region is divided into 6 provinces, which are further divided into 41 rural communes and 6 urban communes. Our study was restricted to rural areas and therefore excluded the urban communes. In addition, four rural communes were excluded due to the implementation of a nutrition-related co-intervention conducted by the Micronutrient Initiative program.

Boucle du Mouhoun is one of the poorest regions of the country [18], most people live in rural areas and work as subsistence farmers producing crops like millet and sorghum. The government is the main health service provider; services are organised under six health districts (corresponding to provinces), each with one regional or district-level hospital and primary health facilities in villages. National policies implemented in public health facilities at the time of our survey included free antenatal care since 2002; the Integrated Management of Childhood Illness (IMCI) strategy since 2003 although a 2011 evaluation found poor adherence to IMCI guidelines [19,20]; and subsidies for delivery and emergency obstetric care since 2006.

### Study design

The data presented in this paper is from the baseline of a before-after cluster-randomised trial. A cross-sectional household survey was designed to select a population-representative sample of women of reproductive age (15 to 49 years) with at least one live birth in the previous 12 months living in rural areas of Boucle du Mouhoun, Burkina Faso. Our study was restricted to mothers of infants under 12 months (rather than 24 months) due to budget limitations.

Women were sampled using a two-stage approach. First, within each rural commune, we randomly selected three villages with probability proportional to size, using the most recent census (2006) as a sampling frame. We then conducted a census of each selected village to identify all eligible mothers within the village. Women were eligible to participate if their infant, aged less than 12 months, was alive and living with them at the time of the survey. Twenty mother-infant pairs (of whom 10 infants were less than 6 months old and 10 were 6 to 11 months old) were sampled per village using stratified simple random sampling.

Our study was powered based on requirements for the cluster-randomised trial, specifically to allow us to detect a 20% point increase in exclusive breastfeeding prevalence in the intervention arm compared to the control arm with a statistical power of 90%, assuming 30% prevalence at baseline, based on the 2010 Demographic and Health Survey [8], and a between-commune coefficient of variation of 0.4. Data from both arms are pooled in this descriptive baseline analysis.

### Data collection

The survey took place between 2^nd^ June and 28^th^ July 2015. Mothers were interviewed using a structured questionnaire, administered using a Trimble Juno SB Personal Digital Assistant, allowing rapid monitoring and feedback of data quality to interviewers. Information was collected on; socio-demographic characteristics, care seeking for childhood illnesses, knowledge, attitude and behaviours related to breastfeeding and complementary feeding, and previous exposure to nutrition-related information. Information on the infant’s diet was collected by asking the mother whether she had given her infant one or more items from a list of 26 liquids and foods. Two recall periods were used: the day and night prior to the interview and the past seven days. The questionnaire was designed based on the Demographic and Health Survey (DHS) questionnaire, as well as that used in the PROMISE trial on exclusive breastfeeding conducted in the Cascades region of Burkina Faso (5, 11). The questionnaire is available online at: https://doi.org/10.17037/DATA.173. The questionnaire was piloted prior to the start of data collection and changes to the wording made where necessary to ensure clarity. Interviewers received two weeks training prior to the start of data collection.

### Outcome definitions

Key infant feeding practices (see below) were defined in line with WHO indicators [21]. Unless otherwise stated, outcomes presented here are based on the 24-hour recall period.

*Exclusive breastfeeding*: the proportion of infants aged <6 months who received only breastmilk during the day and night before the survey. The infant could receive expressed breastmilk, modern medicines including oral rehydration solution, and still be counted as exclusively breastfed. We did not ask mothers directly if the infant was exclusively breastfed; this variable was constructed at the analysis stage on the basis of foods and liquids reported consumed.

*Initiated breastfeeding within 1 hour of birth*: the proportion of currently living infants aged 0–11 months who were put to the breast within one hour of birth.

*Gave colostrum after birth*: the proportion of currently living infants aged 0–11 months who were given colostrum.

*No pre-lacteal feeds*: the proportion of currently living infants aged 0–11 months who did not receive any liquids before the initiation of breastfeeding within the first three days of life.

*Consumed soft*, *semi-solid*, *or solid food*: the proportion of infants aged 6–11 months who received at least one soft, semi-solid or solid food during the day and night before the survey, based on dietary recall.

*Minimum meal frequency*: the proportion of infants aged 6–11 months who were fed the minimum number of times or more during the day and night before the survey. The minimum is defined as two times if the infant is breastfed and 6–8 months-old, three times if the infant is breastfed and 9–11 months-old.

*Minimum dietary diversity*: the proportion of infants aged 6–11 months who received food from four or more different groups (grains, roots and tubers; legumes and nuts; dairy; meat and fish; eggs; vitamin A-rich fruit and vegetables; other fruit and vegetables) during the day and night before the survey. Consumption of any quantity of the food is sufficient unless used only as a condiment.

*Minimum acceptable diet*: the proportion of infants aged 6–11 months who achieved at least both the minimum dietary diversity and minimum meal frequency.

### Statistical analysis

Crude and multivariable logistic regression was used to identify factors predictive of: i) exclusive breastfeeding among infants <6 months; ii) consumption of soft, semi-solid or solid food among infants age 6–11 months. Variables were initially selected for inclusion in the unadjusted models if they had been reported to be relevant in previous studies, and included in the multivariable model if associated with the respective outcome variable p<0.1. Infant age was treated as a continuous variable; data were assessed for evidence of non-linearity. All regression models included random effects to account for clustering within each rural commune; the reliability of the quadrature approximations was assessed. All analyses were conducted using Stata/SE version 14.1.

### Ethical approval

Ethical approval for this study was granted by the National Health Ethic Committee of the Ministry of Health of Burkina Faso (Reference 2015-5-061), the institutional review board of Centre MURAZ (Reference 2015–017) and the London School of Hygiene and Tropical Medicine (Reference 9066). Written informed consent was obtained from all mothers (where the mother was illiterate a thumbprint was used as is standard procedure in Burkina Faso); married mothers aged 15–17 were considered emancipated minors and did not require parental consent. The trial is registered at ClinicalTrials.gov (Reference NCT02435524).

## Results

A total of 2,288 mother-infant pairs participated in this study, 1,173 infants were under 6 months and 1,115 infants were between 6 and 11 months old. Eight eligible women (0.4%) selected for interview were absent or unavailable. No women refused to participate.

The proportion of infants under 6 months reported to be exclusively breastfed during the day and night before the interview was 30% (Table 1). The proportion of infants under 6 months reported to be exclusively breastfed during the past week, had not received any pre-lacteal feeds and had not temporarily stopped breastfeeding for any reason since the birth was 27%. Relatively few mothers (9%) reported initiation of breastfeeding within an hour of birth, although a majority (75%) reported giving their infant colostrum and not giving any pre-lacteal feeds (85%).

**Table 1:** Key feeding indicators among infants under 12 months in Boucle du Mouhoun, N = 2,288.

	Infant age		
	0–5 months	6–11 months	0–11 months
	N = 1,173 (%)	N = 1,115 (%)	N = 2,288 (%)
Initiated breastfeeding within 1 hour of birth	98 (8.4%)	116 (10.4%)	214 (9.4%)
Gave colostrum after birth	890 (75.9%)	817 (73.3%)	1,707 (74.6%)
No pre-lacteal feeds	998 (85.1%)	940 (84.3%)	1,938 (84.7%)
Exclusively breastfed the day and night before the interview	352 (30.0%)	n/a	n/a
Exclusively breastfed throughout the week before the interview	325 (27.7%)	n/a	n/a
Exclusively breastfed since birth*	313 (26.7%)	n/a	n/a
Continued breastfeeding in past day and night	n/a	1,115 (100%)	n/a
Consumed soft, semi-solid or solid food in past day and night	n/a	747 (67.0%)	n/a
Minimum dietary diversity in past day and night	n/a	18 (1.6%)	n/a
Minimum meal frequency in past day and night	n/a	539 (48.3%)	n/a
Minimum acceptable diet in past day and night	n/a	18 (1.6%)	n/a

*Specifically: those exclusively breastfed throughout the week before the interview, who were not given pre-lacteal feeds, and had not temporarily stopped breastfeeding at any point since birth

**Fig 1:**
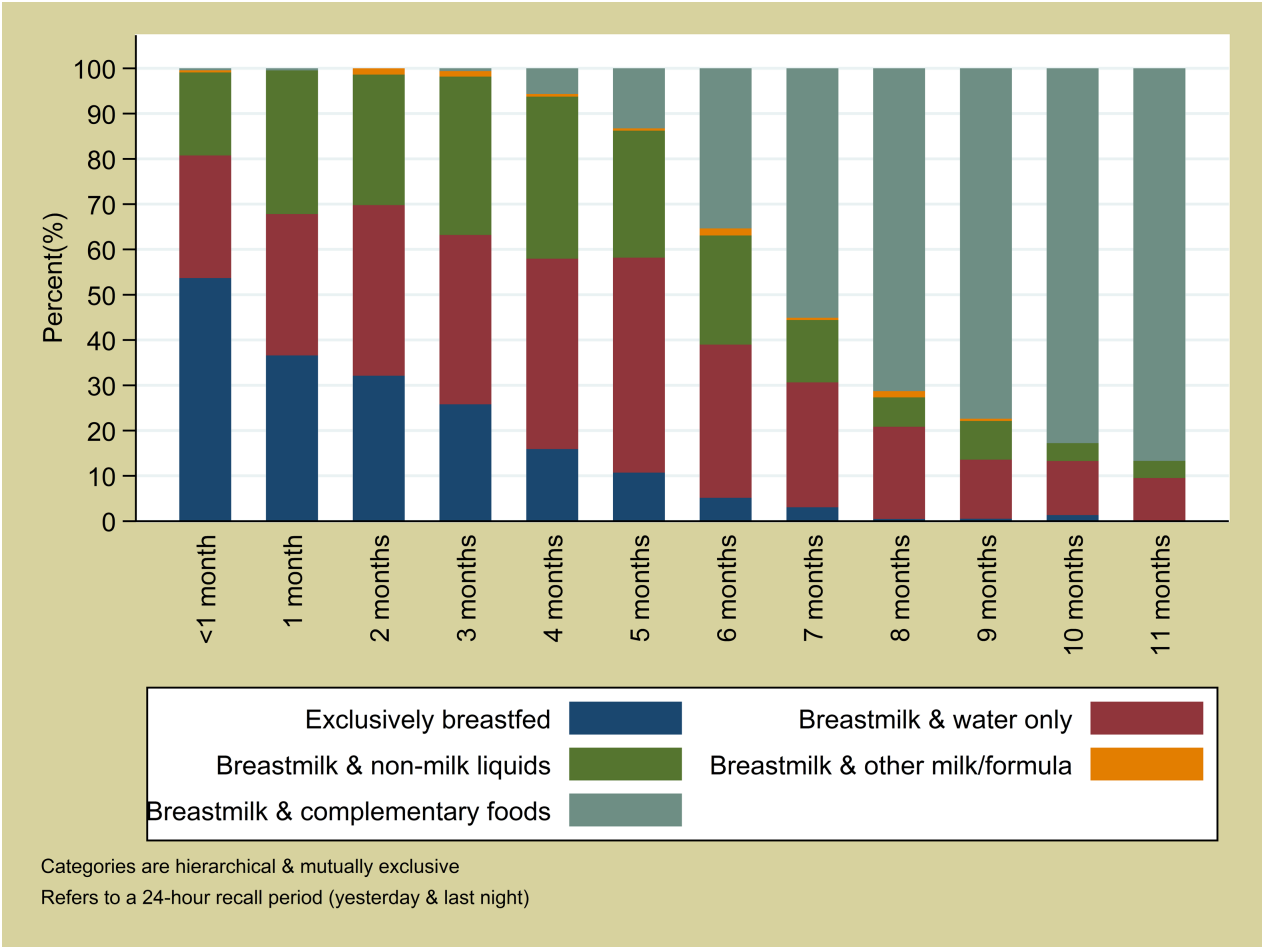
Dietary diversity among infants age 6 to 11 months in Boucle du Mouhoun, N = 1,115.

All infants aged 6 to 11 months were reported to be breastfed during the day and night before the interview and around two thirds (67%) had consumed soft, semi-solid or solid food during the same recall period (54% among infants 6 to 8 months; 82% among infants 9 to 11 months). A negligible proportion (2%) met the WHO criteria for minimum dietary diversity; more infants (48%) met the criteria for minimum meal frequency. The most commonly consumed food group was grains, roots, tubers (32% of infants during the day and the night prior to interview) (Fig 1). Each of the other food groups were consumed by less than 10% of infants. 17% of infants aged 6 to 11 months had consumed food from the grains, roots, tubers group plus at least one other group in the day and night before the interview (27% within last week). There was no evidence that dietary diversity increased with age (p = 0.63), however, the proportion of infants meeting the minimum meal frequency did increase from 28% among infants aged 6 months to 60% among infants aged 11 months (p<0.0001).

**Fig 2:**
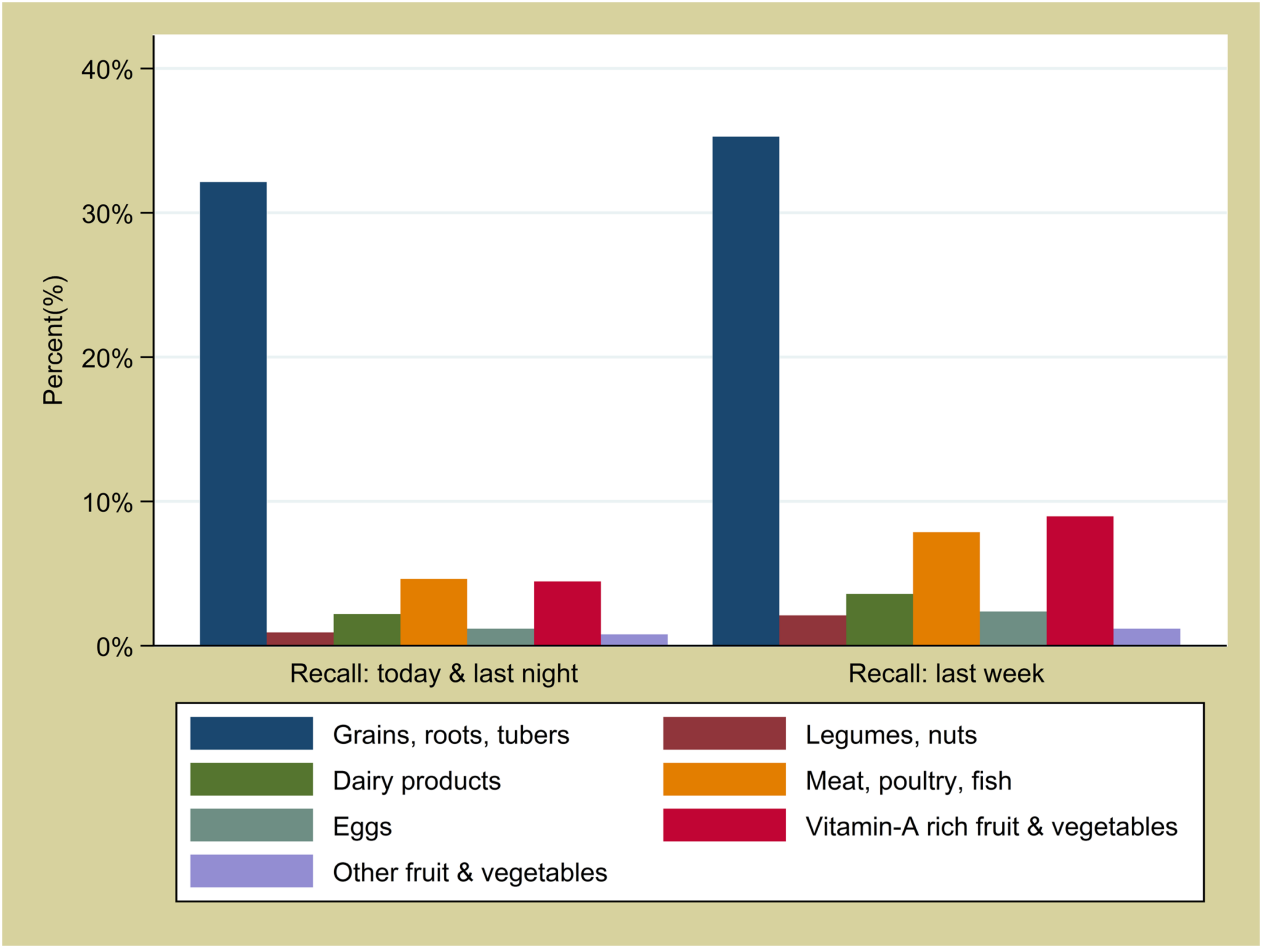
Infant feeding by age in months in Boucle du Mouhoun, N = 2,288.

Fig 2 shows infant feeding patterns by age. The proportion of infants who were exclusively breastfed during the day and night before the interview declined steeply with age, from 54% in infants less than one month old to 11% at 5 months old. At 6 months or older, a persistent minority of 10% or more infants did not receive any soft, semi-solid or solid food the day and night before the interview.

With respect to knowledge and attitudes, around half (51%) of mothers of infants under twelve months correctly stated that an infant should be exclusively breastfed for 6 months after the delivery, but 34% of mothers gave an answer less than this (Table 2). The majority of mothers (89%) thought that an infant should be older than 6 months before complementary foods are introduced. Mothers were generally in agreement with statements to the effect that breastfed babies will have better health. However, nearly three in four mothers (74%) believed that a baby needs to drink water in addition to breastmilk. Only 29% of mothers agreed with the statement that exclusive breastfeeding protects against pregnancy.

**Table 2:** Knowledge and attitudes towards infant feeding among mothers of infants age 0–11 months in Boucle du Mouhoun N = 2,288.

		Infant age		
		0–5 months	6–11 months	0–11 months
		N = 1,173 (%)	N = 1,115 (%)	N = 2,288 (%)
“For how long after delivery should an infant should be uniquely breastfed?”	<3 months	203 (17.3%)	184 (16.5%)	387 (16.9%)
	3–5 months	209 (17.8%)	181 (16.2%)	390 (17.0%)
	6 months	585 (50.0%)	582 (52.1%)	1167 (51.0%)
	More than 6 months	134 (11.4%)	131 (11.7%)	265 (11.6%)
	Don’t know	42 (3.6%)	37 (3.3%)	79 (3.5%)
“At what age should a mother start to give semi-solid or solid food to her infant?”	<6 months	20 (1.7%)	23 (2.1%)	43 (1.9%)
	6 months	93 (7.9%)	96 (8.6%)	189 (8.3%)
	7–8 months	203 (17.3%)	258 (23.1%)	461 (20.2%)
	9 months or older	843 (71.9%)	722 (64.8%)	1565 (68.4%)
	Don’t know	14 (1.2%)	16 (1.4%)	30 (1.3%)
“I would like to know if you agree or disagree with the information I am going to read to you:”				
“Breastfeeding is a good thing for the baby’s health”	Agree	1156 (98.6%)	1108 (99.4%)	2264 (99.0%)
	Disagree	8 (0.7%)	4 (0.4%)	12 (0.5%)
	Don’t know	9 (0.8%)	3 (0.3%)	12 (0.5%)
“If a mother breastfeeds, her baby will have less diarrhoea”	Agree	899 (76.6%)	852 (76.4%)	1751 (76.5%)
	Disagree	208 (17.7%)	202 (18.1%)	410 (17.9%)
	Don’t know	66 (5.6%)	61 (5.5%)	127 (5.6%)
“Cow’s milk is more nourishing for the baby than breastmilk”	Agree	105 (9.0%)	81 (7.3%)	186 (8.1%)
	Disagree	1019 (86.9%)	988 (88.6%)	2007 (87.7%)
	Don’t know	49 (4.2%)	46 (4.1%)	95 (4.2%)
“A baby needs to drink water in addition to breastmilk”	Agree	841 (71.7%)	860 (77.1%)	1701 (74.3%)
	Disagree	302 (25.8%)	232 (20.8%)	534 (23.3%)
	Don’t know	30 (2.6%)	23 (2.1%)	53 (2.3%)
“Whilst a mother is exclusively breastfeeding she can avoid pregnancy”	Agree	361 (30.8%)	305 (27.4%)	666 (29.1%)
	Disagree	615 (52.4%)	650 (58.3%)	1265 (55.3%)
	Don’t know	197 (16.8%)	160 (14.4%)	357 (15.6%)

Predictors of exclusive breastfeeding the day and night before the interview among infants less than 6 months are presented in Table 3. In the multivariable model, there was strong evidence of a negative relationship between exclusive breastfeeding and infant age (AOR: 0.59; 95% CI: 0.53, 0.66; p<0.001). There was strong evidence that infants were more likely to be exclusively breastfed if they were delivered at a health facility compared to home (AOR: 2.63; 95% CI: 1.26, 5.49; p = 0.01), or if they or their mother had attended a postnatal consultation at a facility within a week of the delivery (AOR: 1.50; 95% CI: 1.07, 2.11; p = 0.004). Infants were more likely to be exclusively breastfed if the mother correctly identified six months as the age until which an infant should be exclusively breastfed (AOR: 3.33; 95% CI: 2.38, 4.65; p<0.001). Infants were less likely to be exclusively breastfed if they had been ill in the past two weeks (30% of infants less than 6 months). There was strong evidence of an association between exclusive breastfeeding and mother’s ethnicity, suggesting that social and cultural norms are also likely to be important.

**Table 3:** Predictors of exclusive breastfeeding (EBF) the day and night before the interview among infants 0–5 months in Boucle du Mouhoun N = 1,173.

		N	% EBF	Unadjusted			Adjusted*		
				OR	[95% CI]	p-value	OR	[95% CI]	p-value
Infant age (in months)		1173	-	0.60	[0.54, 0.66]	<0.0001	0.59	[0.53, 0.66]	<0.0001
Infant sex	Female	630	32%	1.00		0.4025			
	Male	543	28%	0.89	[0.67, 1.17]				
Birth order of index infant	First live birth	191	26%	1.00		0.4084			
	2^nd^ or 3^rd^ live birth	395	32%	1.36	[0.89, 2.09]				
	4^th^ to 6^th^ live birth	410	29%	1.25	[0.81, 1.92]				
	7^th^ live birth or higher	177	33%	1.49	[0.90, 2.47]				
Mother’s age	15–24 years	453	28%	1.00		0.3791			
	25–34 years	509	31%	1.18	[0.87, 1.60]				
	35–49 years	211	32%	1.29	[0.87, 1.90]				
Marital status	Married: monogamous	739	27%	1.00		0.3229			
	Married: polygamous	411	34%	1.25	[0.93, 1.67]				
	Single/widow/divorced	23	35%	1.08	[0.42, 2.79]				
Mother’s education	No formal education	890	31%	1.00		0.2534			
	Primary only	202	27%	0.97	[0.66, 1.41]				
	Secondary or higher	81	31%	1.57	[0.91, 2.72]				
Mother earns an income (cash/kind)	No	585	34%	1.00		0.0586	1.00		0.3647
	Yes	588	26%	0.76	[0.57, 1.01]		0.86	[0.62, 1.19]	
Father’s education	No formal education	814	32%	1.00		0.6808			
	Primary only	170	25%	0.74	[0.49, 1.12]				
	Secondary or higher	65	29%	0.98	[0.54, 1.81]				
	Attended, level unknown	80	24%	0.71	[0.40, 1.28]				
	Unknown	21	24%	0.76	[0.26, 2.22]				
	Not currently married	23	35%	0.92	0.36, 2.39]				
Father earns an income (cash/kind)	No	169	34%	1.00		0.4763			
	Yes	975	29%	0.72	[0.48, 1.08]				
	Unknown	6	33%	0.80	[0.13, 5.03]				
	Not currently married	23	35%	0.75	[0.28, 2.06]				
Relative wealth quintile	Poorest	225	37%	1.00		0.0154	1.00		0.0952
	Poorer	230	28%	0.69	[0.45, 1.08]		0.72	[0.44, 1.18]	
	Middle	233	31%	0.95	[0.62, 1.46]		0.91	[0.56, 1.49]	
	Richer	255	22%	0.55	[0.35, 0.86]		0.53	[0.32, 0.88]	
	Richest	230	33%	1.06	[0.68, 1.65]		0.92	[0.55, 1.52]	
Antenatal care for index birth	None	15	7%	1.00		0.2108			
	1–3 visits	432	32%	5.61	[0.67, 47.10]				
	4 or more visits	726	29%	4.92	[0.59, 41.05]				
Place of delivery	Home	91	14%	1.00		0.0019	1.00		0.0101
	Health facility	1082	31%	2.83	[1.47, 5.45]		2.63	[1.26, 5.49]	
Postnatal care within 1 week of delivery	No	721	27%	1.00		0.0468	1.00		0.0186
	Yes	452	34%	1.36	[1.00, 1.85]		1.50	[1.07, 2.11]	
Infant has been ill in last 15 days ^#^	No	816	34%	1.00		<0.0001	1.00		0.0043
	Yes	357	20%	0.39	[0.28, 0.54]		0.60	[0.40, 0.84]	
Mother correctly identifies 6 months as the time until which an infant should be EBF	No	588	20%	1.00		<0.0001	1.00		<0.0001
	Yes	585	40%	2.89	[2.16, 3.88]		3.33	[2.38, 4.65]	
Mother’s ethnic group	Bobo	131	46.6%	1.00		<0.0001	1.00		0.0002
	Bwaba	144	39.6%	0.97	[0.57, 1.66]		0.96	[0.51, 1.79]	
	Dafing	275	13.5%	0.26	[0.14, 0.47]		0.23	[0.12, 0.46]	
	Mossi	252	41.7%	0.83	[0.49, 1.41]		0.77	[0.42, 1.43]	
	Peulh	77	31.2%	0.52	[0.26, 1.04]		0.44	[0.20, 0.99]	
	Samo	154	25.3%	0.40	[0.20, 0.78]		0.36	[0.16, 0.78]	
	Other	140	20.7%	0.41	[0.21, 0.80]		0.47	[0.22, 1.01]	

* *Adjusted for all other variables in in the adjusted column*

^#^ *Had one or more symptoms: fever, cough, rapid breathing or difficulties breathing, diarrhoea*

With respect to predictors of consuming soft, semi-solid or solid food the day and night before the interview among infants aged 6 to 11 months, there was strong evidence of a positive association with infant age (Table 4). There was weak evidence that mothers who earned an income were more likely to have fed their infant soft, semi-solid or solid food the day before the interview (AOR: 1.33; 95% CI: 1.00, 1.77; p = 0.048). There was also weak evidence that infants born in a health facility were more likely to have been fed complementary foods compared to those born at home (AOR: 1.59; 95% CI: 1.00, 2.51; p = 0.048).

**Table 4:** Predictors of consuming soft, semi-solid or solid food (SSS) the day and night before the interview among infants 6–11 months in Boucle du Mouhoun N = 1,115.

		N	% SSS	Unadjusted			Adjusted*		
				OR	[95% CI]	p-value	OR	[95% CI]	p-value
Infant age (in months)		1115	-	1.74	[1.58, 1.91]	<0.0001	1.73	[1.57, 1.90]	<0.0001
Infant sex	Female	569	68%	1.00		0.3580			
	Male	546	66%	0.89	[0.69, 1.14]				
Birth order of index infant	First live birth	203	67%	1.00		0.8354			
	2^nd^ or 3^rd^ live birth	323	69%	1.11	[0.75, 1.62]				
	4^th^ to 6^th^ live birth	395	66%	0.95	[0.66, 1.37]				
	7^th^ live birth or higher	194	66%	1.00	[0.65, 1.54]				
Mother’s age	15–24 years	396	67%	1.00		0.4642			
	25–34 years	477	69%	1.07	[0.80, 1.43]				
	35–49 years	242	64%	0.87	[0.62, 1.23]				
Marital status	Married: monogamous	696	67%	1.00		0.2905			
	Married: polygamous	389	67%	1.00	[0.76, 1.31]				
	Single/widow/divorced	30	80%	2.09	[0.83, 5.26]				
Mother’s education	No formal education	828	65%	1.00		0.0804	1.00		0.1097
	Primary only	204	74%	1.48	[1.04, 2.11]		1.40	[0.95, 2.05]	
	Secondary or higher	83	70%	1.21	[0.74, 2.00]		1.49	[0.86, 2.57]	
Mother earns an income (cash/kind)	No	531	62%	1.00		0.0013	1.00		0.0484
	Yes	584	71%	1.53	[1.18, 1.98]		1.33	[1.00, 1.77]	
Father’s education	No formal education	760	66%	1.00		0.1893			
	Primary only	175	66%	0.97	[0.68, 1.39]				
	Secondary or higher	62	79%	1.89	[1.00, 3.59]				
	Attended, level unknown	73	66%	0.96	[0.57, 1.61]				
	Unknown	15	53%	0.61	[0.22, 1.75]				
	Not currently married	30	80%	2.13	[0.85, 5.36]				
Father earns an income (cash/kind)	No	166	65%	1.00		0.1970			
	Yes	910	67%	1.14	[0.80, 1.64]				
	Unknown	9	89%	4.48	[0.53, 37.63]				
	Not currently married	30	80%	2.36	[0.90, 6.23]				
Relative wealth quintile	Poorest	231	66%	1.00		0.8480			
	Poorer	228	67%	1.03	[0.69, 1.53]				
	Middle	224	67%	1.06	[0.71, 1.58]				
	Richer	204	65%	0.93	[0.62, 1.40]				
	Richest	228	70%	1.18	[0.78, 1.76]				
Antenatal care for index birth	None	13	62%	1.00		0.1766			
	1–3 visits	453	64%	1.00	[0.31, 3.18]				
	4 or more visits	649	69%	1.28	[0.40, 4.05]				
Place of delivery	Home	117	56%	1.00		0.0123	1.00		0.0476
	Health facility	998	68%	1.68	[1.12, 2.53]		1.59	[1.00, 2.51]	
Postnatal care within 1 week of delivery	No	704	68%	1.00		0.1455			
	Yes	411	66%	0.81	[0.62, 1.07]				
Infant has been ill in last 15 days ^#^	No	629	66%	1.00		0.2130			
	Yes	486	69%	1.18	[0.91, 1.53]				
Mother correctly identifies 6 months as the time from when an infant should be SSS	No	1,019	66%	1.00		0.1696			
	Yes	96	75%	1.41	[0.86, 2.31]				
Mother’s ethnic group	Bobo	147	66.7%	1.00		0.8355			
	Bwaba	145	67.6%	1.18	[0.70, 1.97]				
	Dafing	263	63.9%	0.98	[0.61, 1.58]				
	Mossi	214	66.8%	1.08	[0.67, 1.75]				
	Peulh	77	66.2%	1.20	[0.64, 2.27]				
	Samo	136	69.9%	1.21	[0.69, 2.14]				
	Other	133	70.7%	1.42	[0.80, 2.54]				

* *Adjusted for all other variables in the adjusted column*

^#^ *Had one or more symptoms: fever, cough, rapid breathing or difficulties breathing, diarrhoea*

Despite all mothers reporting contact with a health worker at one or more time point during or since the index pregnancy (99% attended ANC at least once; 91% delivered in a health facility; 38% had attended postnatal care within a week of delivery; 99% took the infant to be weighed at least once; 89% took the infant to a vaccinations at least once; 18% had taken the infant to a health facility due to illness in the past 15 days), only 42% reported receiving information or advice about breastfeeding from a health worker (Table 5).

**Table 5:** Sources of information on breastfeeding among mothers of infants 0–11 months in Boucle du Mouhoun N = 2,288.

	Infant age		
	0–5 months	6–11 months	0–11 months
	N = 1,173 (%)	N = 1,115 (%)	N = 2,288 (%)
Mother reports she was given (any) advice about breastfeeding by a relative since the delivery:			
Given advice by mother	189 (16.1%)	179 (16.1%)	368 (16.1%)
Given advice by mother-in-law	121 (10.3%)	93 (8.3%)	214 (9.4%)
Given advice by other female relative	64 (5.2%)	67 (3.3%)	131 (5.7%)
Given advice by husband	4 (0.3%)	3 (0.3%)	7 (0.3%)
Given advice by other male relative	7 (0.6%)	5 (0.5%)	12 (0.5%)
**Given (any) advice by one or more relatives**	**252 (21.5%)**	**251 (22.5%)**	**503 (22.0%)**
**Given advice to exclusively breastfeed by one or more relative**	**131 (11.2%)**	**137 (12.3%)**	**268 (11.7%)**
Mother reports she was given advice about breastfeeding by a health worker during antenatal care or since the delivery:			
During antenatal care	217 (21.1%)	284 (25.5%)	531 (23.2%)
At the time of delivery	300 (25.6%)	293 (26.3%)	593 (25.9%)
During a postnatal consultation within a week of delivery	149 (12.7%)	145 (13.0%)	294 (12.8%)
During a consultation for infant weighing	112 (9.6%)	217 (19.5%)	329 (14.4%)
During a consultation for vaccination	125 (10.7%)	153 (13.7%)	278 (12.2%)
During a consultation due to illness of the infant in last 15 days	32 (2.7%)	38 (3.4%)	70 (3.1%)
**Given advice at one or more time point**	**463 (39.5%)**	**501 (44.9%)**	**964 (42.1%)**
Mother reports that she has been visited at home by a community health worker to advise on breastfeeding since the delivery	7 (0.5%)	13 (1.2%)	20 (0.9%)
Mother reports that she has heard information about breastfeeding in a group discussion in a public place	167 (14.2%)	246 (22.1%)	413 (18.1%)
Mother reports that she has heard information about breastfeeding on the radio	207 (17.6%)	244 (21.9%)	451 (19.7%)

Only 22% of interviewed mothers reported receiving information or advice from a relative and, among them, around half said that the advice given was to breastfeed exclusively; other advice frequently related to the preparation of tisanes for the baby or remedies for painful breasts. The woman’s mother (16%), followed by her mother-in-law (9%), were the relatives most likely to give advice. Around one in five mothers reported that they had heard information about breastfeeding either in a public place (18%) or on the radio (20%).

## Discussion

With the exception of continued breastfeeding, infant feeding indicators in Boucle du Mouhoun are poor. Concurrently, we found that many mothers have poor knowledge in key areas and a minority reported receiving information and advice from a health worker. Whilst there was strong evidence of a positive association between having correct knowledge of the recommended time period for exclusive breastfeeding and exclusive breastfeeding in practice (an exclusive breastfeeding rate of 40% among mothers with correct knowledge versus 20% with incorrect knowledge), six out of ten mothers with correct knowledge still did not breastfeed exclusively. Clearly other barriers to optimal infant feeding practices exist in Boucle du Mouhoun. Our model found health facility delivery and early postnatal care to be significant predictors of exclusive breastfeeding. There was also strong evidence of an association with ethnic group, suggesting social and cultural norms may play an important role. In-depth qualitative work would be useful to understand these norms further.

In our study, just under a third of infants under 6 months of age were exclusively breastfed. Whilst this is similar to the figure for Boucle du Mouhoun reported in the most recent DHS [8], it nonetheless represents a lower prevalence than reported in other similar settings: recent data suggests that overall in low-income countries prevalence of exclusive breastfeeding is 47% [3]. We noted a firmly held belief among many mothers that a baby needs to drink water in addition to breastmilk. A contributing factor to this belief may be the hot and semi-arid climate in Boucle du Mouhoun, which has a monthly maximum temperature ranging between 35 degrees Celsius in August and 44 degrees Celsius in May [22]. This finding is of particular concern in a setting where 29% of rural households principally get their water from an unimproved source [8]. Qualitative work from Zambia has described how mothers can have a fear of producing insufficient breastmilk and not providing enough energy to satisfy their child, and a perceived need for an infant to get used to other sources of nutrition early in case the mother becomes ill or dies [23]. Further qualitative work specific to social norms surrounding infant feeding in the context of Burkina Faso is needed. While infants were less likely to be exclusively breastfed if they had been ill in the past two weeks, the direction of causation cannot be assessed in this cross-sectional study.

Complementary feeding indicators among infants aged 6 to 11 months were particularly poor. Less than 2% of infants in this age group had a minimum acceptable diet, according to WHO criteria. Around a third of infants aged 6 to 11 months had only received liquids, often breastmilk and water, the day and night before the interview; whilst around 10% of infants even at 11 months old were receiving breastmilk and water only. Receiving inadequate levels of nutrition during early stages of development is clearly a cause for concern; the WHO has set a global target of reducing by 40% the number of stunted under-five children by 2025 [24]. National-level data from the most recent DHS show that among infants aged 6 to 11 months, 93% were anaemic (83% moderate or severe); 13% of infants 6 to 8 months and 25% of infants 9 to 11 months were stunted; 34% of infants 6 to 8 months and 30% of infants 9 to 11 months were wasted; and 26% of infants 6 to 8 months and 37% of infants 9 to 11 months were underweight [8]. Our survey took place during the rainy season in Burkina Faso, and it is possible that dietary diversity may improve at other times of year; nonetheless the issue is still one of importance that needs to be addressed. The last DHS—conducted between May and December—also found similar results [8]. In contrast to the findings on exclusive breastfeeding, poor knowledge mirrored reported practice. Of great concern was the late age at which many mothers believed complementary foods should be introduced: only 8% of mothers correctly identified 6 months as the age at which an infant should start to be fed complementary foods, whilst one in five mothers thought that this should take place at age seven or eight months, and nearly seven in ten mothers said that this should happen at nine months or older.

Our study was a population-representative survey with a substantial sample size. We were able to collect information on knowledge relating to infant feeding, covering the spectrum of behaviours, knowledge and attitudes. Nonetheless, our study also had some limitations. First, our outcomes were based on mothers’ self-report. It is possible that some mothers may have tended to over-report exclusive breastfeeding in order to create a positive impression with the interviewer. Biological validation techniques exist but are expensive due to the need for laboratory facilities [25]. In order to mitigate the risk of over-reporting, mothers were not directly asked whether or not they exclusively breastfed their infant and were instead asked a detailed set of questions about everything the infant had eaten or drunk during the recall period. Second, we did not collect anthropometric data on infant weight or length so are unable to report on these variables. Third, our data were collected during a two-month period (June and July) and it is possible that our findings may have been influenced by seasonality in food availability. Finally, our study population is mothers and their infants living in rural areas within one region of Burkina Faso. We are not necessarily able to generalise these findings to other parts of the country; it is noteworthy that differences between ethnic groups were important predictors of infant feeding patterns.

Given poor practices and low levels of knowledge, targeted interventions are needed to improve infant nutrition in this region. Gaps in the availability and quality of postpartum care for mothers and infants were recently identified as a priority for intervention by stakeholders in rural Burkina Faso [26]. It is interesting to note that, despite national guidelines stating that all mothers should be given advice, support and counselling regarding infant feeding during routine care [27–29], less than half of all mothers in our study reported being given information or advice on breastfeeding by a health worker at any time during the index pregnancy, delivery or postnatal period. A high proportion of women now use antenatal care, facility-based delivery care or postnatal care in Burkina Faso [30] and indeed all women in our study had accessed health services at least once during pregnancy or the postpartum period. Prior to discharge and during the early neonatal period is the ideal time to provide advice and support for infant feeding, therefore increased attention should therefore be paid to increasing the quality of care within facilities, and ensuring that existing guidelines concerning counselling on infant feeding are adhered to. Given the very poor complementary feeding practices observed in this study; attention should also be paid to this issue, including developing future interventions.
